# Antibody Detection, Isolation, Genotyping, and Virulence of *Toxoplasma gondii* in Captive Felids from China

**DOI:** 10.3389/fmicb.2017.01414

**Published:** 2017-07-25

**Authors:** Yu-Rong Yang, Yong-Jie Feng, Yao-Yao Lu, Hui Dong, Tong-Yi Li, Yi-Bao Jiang, Xing-Quan Zhu, Long-Xian Zhang

**Affiliations:** ^1^Department of Basic Veterinary, College of Animal Science and Veterinary Medicine, Henan Agricultural University Zhengzhou, China; ^2^Zhengzhou Zoo Zhengzhou, China; ^3^State Key Laboratory of Veterinary Etiological Biology, Key Laboratory of Veterinary Parasitology of Gansu Province, Lanzhou Veterinary Research Institute, Chinese Academy of Agricultural Sciences Lanzhou, China

**Keywords:** *Toxoplasma gondii*, captive felids, public health, epidemiology, isolation, genotype, virulence, oocysts

## Abstract

The felids are the only definitive hosts of *Toxoplasma gondii*, which could excrete oocysts into the environment and provide an infection source for toxoplasmosis in various warm-blooded animal species, particularly the captive felids that live close to human communities. The infection rate of the captive felids is a perfect standard in detecting the presence of *Toxoplasma gondii* oocysts in the environment. In this study, sera or tissue samples from zoo (1 young tiger, 2 adult tigers, 6 young lions), farm (10 masked palm civets), and pet hospital (28 cats) from Henan Province (China) were collected. The sera (*n* = 47) were tested for immunoglobulin G (IgG) antibodies against *T. gondii* by using modified agglutination test (MAT), whereas the hearts tissue (*n* = 40) were bioassayed in mice to isolate *T. gondii* strains. The genotype was distinguished by using PCR-RFLP of 10 loci (SAG1, SAG2, SAG3, GRA6, BTUB, L358, c22-8, PK1, c29-2, and Apico). The detection rate for the *T. gondii* antibody in captive felids was 21.3% (10/47). One viable *T. gondii* strain (TgCatCHn4) was obtained from a cat heart tissue, and its genotype was ToxoDB#9. The oocysts of ToxoDB#9 were collected from a *T. gondii*-free cat. The virulence of TgCatCHn4 was low and no cysts were detected in the brain of mice at 60 days post-inoculation. The finding of the present study suggested a widespread exposure of *T. gondii* for felids in Henan Province of central China, particularly those from the zoological gardens and homes. ToxoDB#9 was the predominant strain in China. Preventive measures against *T. gondii* oocyst contamination of various components of the environment should thus be implemented, including providing pre-frozen meat, well-cooked cat food, cleaned fruits and vegetables, monitoring birds and rodents, inactive *T. gondii* oocysts in felids feces, and proper hygiene.

## Introduction

*Toxoplasma gondii* infects warm-blooded animals, including birds, livestock, humans, and felids. *T. gondii* induces lymphadenopathy, retinochoroiditis, encephalitis, abortion, and death in immunocompromised individuals (Hide, 2016). Felids are important in the epidemiology of toxoplasmosis for these are the only definitive hosts that can shed environmentally resistant oocysts (Dubey, 2010). One *T. gondii* free domestic or wild felid could shed millions of oocysts after ingestion raw meat that contain *T. gondii* cyst, and the oocysts could survive in soil for years. Furthermore, oocysts are transported via freshwater runoff into the ocean and thus may be a threat to the marine ecosystem, particularly marine mammals (Vanwormer et al., 2016). Approximately 16% of beach cast carcasses of sea otters in California was due to *T. gondii* (Miller et al., 2004).

There are 37 species of felids around the world, with China hosting at least 20 species, with most of these endangered (Johnson et al., 2006), and an estimated 53 millions are domestic cats. The number of wild felids in China has declined due to the destruction of natural habitats in the advent of agricultural development and economic expansion. Most existing felids are artificially bred in farms or kept in zoological parks.

The seroprevalence of *T. gondii* in felids from zoo (84.2%) (Zhang et al., 2000) and masked palm civets (27.6%) from a farm (Hou et al., 2016) was evaluated by using a modified agglutination test (MAT). The prevalence of toxoplasmosis was about 50% in cats from China, and viable *T. gondii* strains had been isolated from tissues or fecal samples of domestic cats (Dubey et al., 2007; Zhou et al., 2009; Chen et al., 2011; Qian et al., 2012; Wang et al., 2013; Li et al., 2015; Yang et al., 2015). The epidemiological role of felids in toxoplasmosis still needs to be fully established. Accordingly, the present study aimed to determine the frequency of *T. gondii* antibodies in felids from zoos, farms, and pet hospitals, in an attempt to isolate viable *T. gondii*.

## Materials and methods

### Sample collection

**Figure 1:**
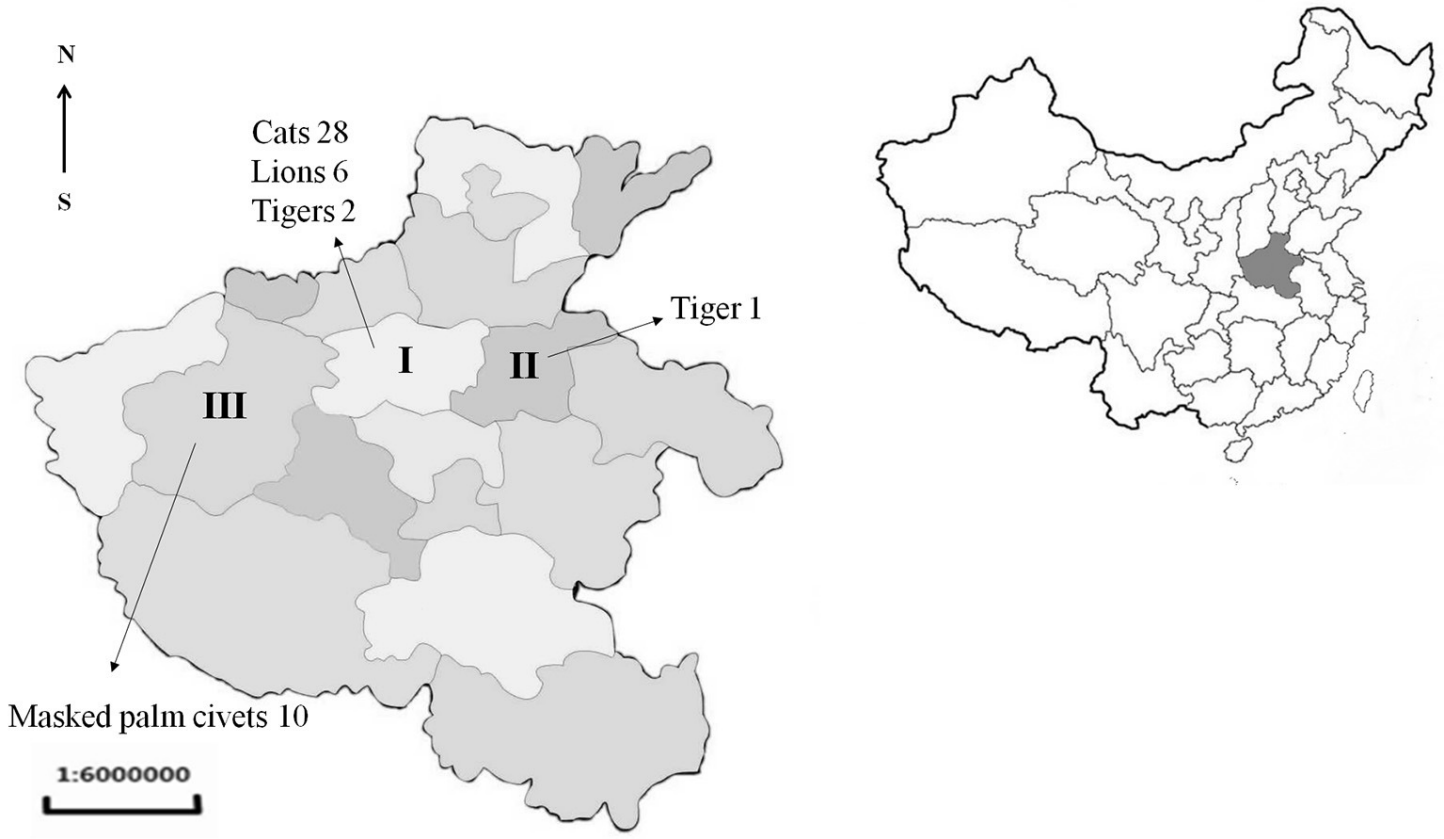
Location and number of samples received from Henan province of China. I, Zhengzhou; II, Kaifeng; III, Luoyang.

From 2015 to 2017, the sick or dead felids due to idiopathic reasons were gathered from zoos, farms, and pet hospitals in Henan Province (33°N, 113.30°E), which is located in central China and has a humid and subtropical climate. Tissue or serum samples were collected from these animals, placed in a cooler box, and then transported to the Laboratory of Veterinary Pathology of Henan Agricultural University (Zhengzhou, Henan Province, China) for pathological diagnosis (Table 1, Figure 1). A total of 21 batches were obtained, including 28 pet cats (*Felis catus*) (28 hearts, 28 sera), 10 masked palm civets (*Paguma larvata*) (7 hearts, 10 sera), 3 tigers (*Panthera tigris altaica*) (2 hearts, 3 sera), and 6 lions (*Panthera leo*) (3 hearts, 6 sera). Feline tissue and serum samples were stored at 4°C and tested within 1 week. Most pet cats were fed commercial cat food. The masked palm civets were fed cooked chicken giblets and cleaned fruit and vegetables in the farm. Tigers and lions were fed raw chicken and pork ribs in the zoo.

**Table 1:** Seroprevalence and isolation of *Toxoplasma gondii* from felids in Henan Province, China.

**Sample received date**	**Location^a^**	**Species, Source**	**Alive/dead**	**Age (m)**	**Sex (F/M)^b^**	**No. of serm**	**No. of hearts**	**Titers of MAT**	**% (Positive No. /Test No.)**	**Isolation obtained from mouse sample^c^**
Mar 26, 2015	I	Domestic cat, Pet hospitals	Dead	24	1 M	1	1	<1:25	7.1 (2/28)	0/1
Mar 31, 2015				3	1 F, 1 M	2	2	1,600–3,200		1^d^/2
April 5, 2015				3	2 F, 4 M	6	6	<1:25		0/6
May 24, 2015				2-3	3 F, 4 M	7	7			0/7
Sep 2, 2015				3	3 F, 1 M	4	4			0/4
Oct 23, 2015				3	2 F, 2 M	4	4			0/4
Nov 11, 2015				3-4	2 M	2	2			0/2
Nov 15, 2016				12	2 M	2	2			0/2
Jan 5, 2016	III	Masked palm civet, Farm	Dead	6	1 F	1	1	<1:25	0(0/10)	0/1
Jan 19, 2016				2	2 F	2	2			0/2
Jan 25, 2016				7	2 F, 1 M	3	3			0/3
April 6, 2016				10	1 F	1	1			0/1
April 17, 2016			Alive	12	3 F	3	0			−
Jan 12, 2016	I	Lion, Zoo	Alive	12	unknown	2	0	1,600	100 (6/6)	−
Sep 18, 2016			Dead	11	1 F	1	1	800		0/1
Sep 25, 2016				12	1 F	1	1	1,600		0/1
Sep 29, 2016				12	1 M	1	1	1,600		0/1
Feb 14, 2017				18	1 F	1	0	400		−
July 2, 2015	I	Tiger, Zoo	Dead	1 day	1 M	1	1	<1:25	66.7 (2/3)	0/1
Dec 30, 2016	II			≥120	1 M	1	1	50		0/1
Feb 14, 2017	I		Alive	12	1 F	1	0	400		−
Total						47	40		21.3 (10/47)	1/40

a *Sampling city in Figure 1*.

b *Number of females/number of males*.

c *Number of positive groups/number of inoculated groups*.

d *The gender of cat which T. gondii was isolated was female*.

### Serological examination by MAT

Forty-seven serum samples from captive felids were serologically assessed for antibodies against *T. gondii* by using MAT at a dilution of 1:25 to the final maximum titer (Dubey and Desmonts, 1987). The positive and negative controls, and the blank were included in each microtiter plate. Whole formalin fixed *T. gondii* tachyzoites was kindly provided by Dr. J. P. Dubey (ARS, USDA), which was obtained from Kerafast company (Catalog No. EH2002).

### Isolation of viable *T. gondii* from felid hearts by using a bioassay in mice

Specific-pathogen-free Swiss mice were supplied by the Zhengzhou University Laboratory Animal Center (China), mouse grant No. was 41003100000236. The hearts of captive felids (*n* = 40) were weighed, washed, homogenized, digested in pepsin, centrifuged, and neutralized, and the homogenate was inoculated subcutaneously into five outbred Swiss mice (healthy, weight ≥ 25 g, ages ≥ 8 weeks) following the description by Dubey (2010) and Yang et al. (2017). Pepsin was purchase from Sigma (Product No. P7012). The seronegative (MAT titer < 25) tissues in each batch were pooled prior to digestion, whereas the seropositive tissues (MAT titer ≥ 25) were digested and bioassayed individually. The tachyzoites or tissue cysts of *T. gondii* were examined in tissue imprints of lungs and brains of mice. The surviving mice were bled on day 60 post-inoculation (DPI), and the *T. gondii* serum antibodies were tested by using MAT with 1:25 and 1:200 dilutions. The mice were sacrificed at 61 DPI, and squash of their brains were prepared and examined for tissue cysts. All brains of the mice were homogenized and sub-passaged into new groups of mice subcutaneously. The mice were determined to be infectived with *T. gondii* when tachyzoites or tissue cysts were detected or showed seropositive MAT results.

### *T. gondii* oocyst collection and virulence evaluation

*Toxoplasma gondii*-free cats were fed tissues of *T. gondii*-positive Swiss mice that were inoculated with infected feline derived homogenates. Their feces were collected daily for 3 weeks and stored at 4°C to prevent oocyst sporulation. The oocysts were collected from the feces as previously reported (Dubey, 2010). Briefly, fecal samples were washed in water, gauze filtered, and then floated in 33% sucrose solution. The supernatant was then collected and kept in 2% sulfuric acid in 25°C for sporulation. Virulence of the *T. gondii* isolated from the felid was evaluated in Swiss mice. Sporulated oocysts were neutralized, counted in a disposable hemocytometer, and diluted 10-fold from 10^−1^ to10^−7^ to reach an end-point of < 1 oocyst. Then, < 1, 10^0^, 10^1^, 10^2^, 10^3^, 10^4^, and 10^5^ oocysts were orally administered to five Swiss mice for each dilution. The clinical symptoms and mortality were recorded daily, and 60 days later, all surviving mice were bled and tested for IgG antibodies to *T. gondii* by the MAT using titers between 1:25 and 1:200. The mice were sacrificed at 61 DPI, and all the tissues were fixed in 10% (v/v) neutral buffered formalin. The tissues were processed by using routine histological processing techniques, and embedded in paraffin. Paraffin sections (5 μm in thickness) of the samples were prepared and stained with hematoxylin and eosin (H&E) and immunohistochemistry (IHC). The primary antibody was rabbit anti-*T. gondii* polyclonal antibody, and tissue sections of a ToxoDB#216 *T. gondii*-infected model was used as positive control (kindly provided by Dr. Dubey, ARS, USDA). A mouse-specific HRP/DAB IHC detection kit was purchased from Abcam (ab64264). All mice were considered infected when antibodies of *T. gondii* or parasites were detected in their sera or tissues.

### Cell cultivation and genotyping

Tissue (brain, heart, and tongue) homogenates of *T. gondii*-positive mice were seeded into *Vero* cell cultures (RPMI 1640, 10% fetal bovine serum, 37°C, 5% CO_2_), refresh the culture fluid after 1 h then the cell culture fluid was replaced twice a week (Dubey, 2010). The number of cysts in the brains of the mice was counted under a microscope using the method described by Dubey et al. (2012). DNA was extracted from *T. gondii* oocysts which were shed by cat. A commercial DNA extraction kit (Tiangen Biotec Company, DP304) was used in DNA extraction. *T. gondii* genotype was distinguished by PCR-RFLP of 10 genetic markers (SAG1, SAG2, SAG3, GRA6, BTUB, L358, PK1, c22-8, c29-2, and Apico) (Su et al., 2010). References of *T. gondii* DNA was included in all batches.

### Ethics approval

The study was approved by the Institutional Animal Use Protocol Committee of the Henan Agricultural University, China. The Beijing Association for Science and Technology (Approval SYXK [Beijing] 2007-0023) approved the protocol used in this study. All captive felids and mice were handled in strict accordance with the good animal practices of the Animal Ethics Procedures and Guidelines of the People's Republic of China.

### Statistical analysis

Statistical analysis was performed by using Graph Pad Prism 7.0 software, which was developed by GraphPad Software Inc., San Diego, CA, USA. The chi-square test or Fisher's exact test was used in data analysis. *P* < 0.05 was considered statistically significant.

## Results

Modified agglutination test (MAT) analysis indicated that the seropositive rate of IgG antibodies to *T. gondii* was 21.3% (10/47) in captive felids, 7.1% (2/28) in domestic cats, 0% (0/10) in masked civets, 66.7% (2/3) in tigers, and 100% (6/6) in lions. The titer of *T. gondii* in seropositive felids was high (50-3, 200) (Table 1). Seropositivity rates varied with respect to the source of the felids. The seroprevalence of *T. gondii* in felids from zoos (88.9%, 8/9) was significantly higher than those from domestic felids (7.1%, 2/28) and farms (0%, 0/10) (*P* < 0.01). In the present study, 6.9% (2/29) of felids ≤ 6 months old were seropositive for *T. gondii*, whereas 44.4% (8/18) of those > 6 months old were seropositive. The positive rate of *T. gondii* in adult felids was significantly higher compared to that in juveniles (*P* = 0.0037). Female felids (20.8%, 5/24) were more susceptible to *T. gondii* than males (14.3%, 3/21), although this difference was not significant (Table 2).

**Table 2:** Effect of age, sex, and sample sources of felids on *Toxoplasma gondii*- positive rates.

**Factor**	**Sample type**	**Sample number**	**Number of positive samples and rate (%)**	***P*-value**
Age^*^	≤6 month	29	2(6.9)	
	>6 month	18	8(44.4)	0.0037
Sex	Female	24	5(20.8)	
	Male	21	3(14.3)	0.7050
Sample sources^*^	Farm	10	0(−)	1.000
	Pet hospitals	28	2(7.1)	
	Zoo	9	8(88.9)	0.0001

* *P-value < 0.05 by two-tailed chi-square tests for T. gondii in both age and sample sources groups*.

**Figure 2:**
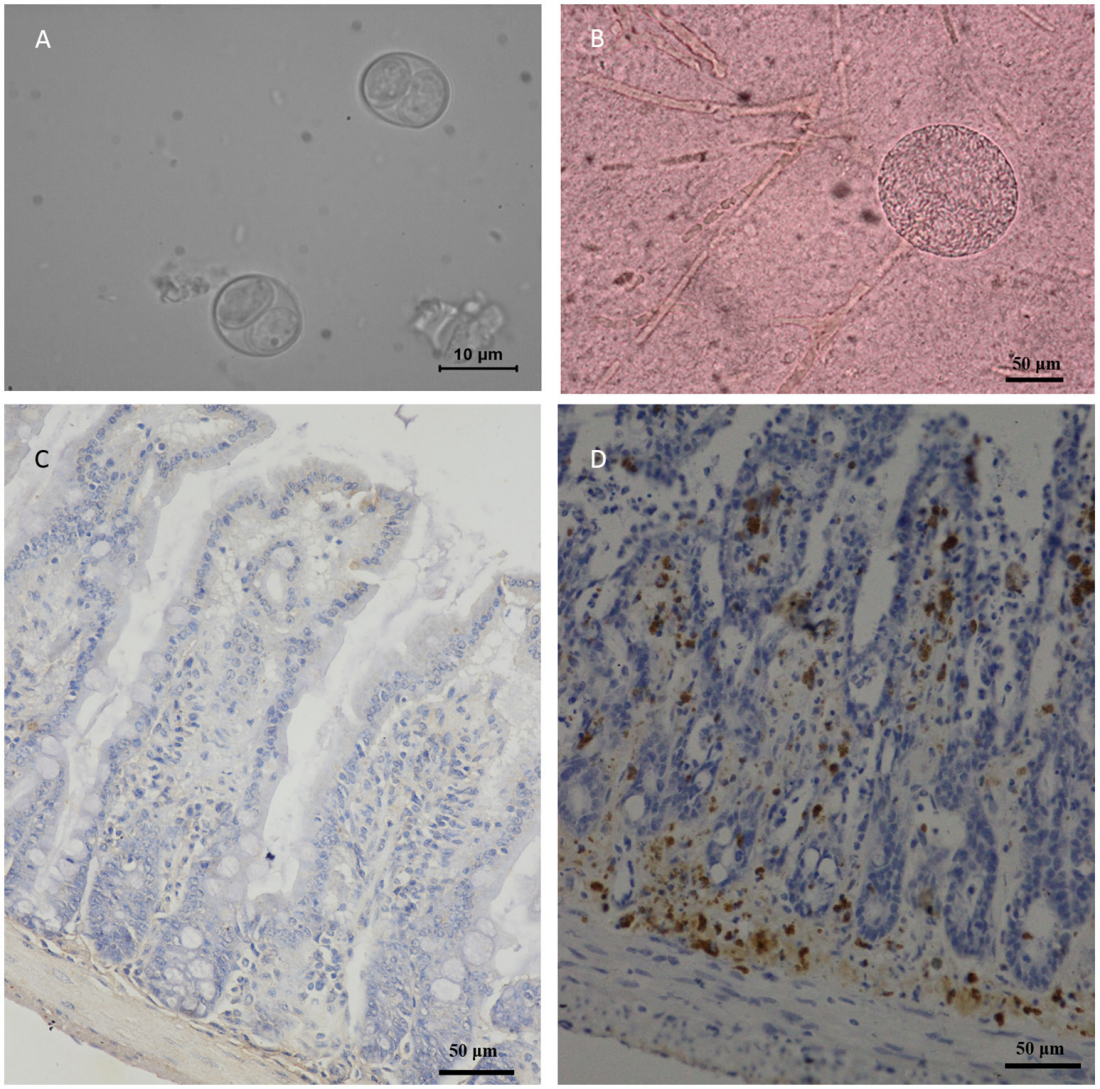
Morphology of TgcatHn4 strain *T. gondii* and the pathological changes in the ileum of Swiss mice. **(A)**, *T. gondii* oocysts were detected in cat fecal samples, 6 DPI, unstained; **(B)**, Tissue cysts of *T. gondii* in mouse brain, 138 DPI, squash, unstained; **(C)**, Ileal lesions were not found, mouse, 60 DPI, *T. gondii* IHC staining; **(D)**, Positive control, ToxoDB#216, ileum, mouse, 6 h post-inoculation, *T. gondii* IHC staining.

A total of 40 felids heart homogenates were individually bioassayed in mice. All mice inoculated with animal tissues remained alive at 60 DPI. *T. gondii* antibodies were only detected in one group of mice at 60 DPI. Viable *T. gondii* were isolated from this positive group (Table 1). The tissues of *T. gondii*-positive mice were sub-inoculated into mice, fed to *T. gondii*-free cat, and seeded onto cell cultures for the propagation of parasites. This isolate *T. gondii* strain was successfully propagated in mice, and *T. gondii* occysts were detected in the feces of cat (Figure 2A); however, it showed a relatively low growth rate in cell culture during 6 month observation. The present study was not successful in isolating *T. gondii* from tigers, lions, MAT seronegative masked palm civets, and cats.

Oocysts of the *T. gondii* strain TgcatHn4 were collected from the feces of *T. gondii*-free cat at days 6–11 after feeding it with mouse tissues. DNA was isolated from *T. gondii* oocysts. Genotype indicated that the isolate from the cat heart was of the ToxoDB genotype #9. The mice were asymptomatic, no deaths were observed, and above 10^3^ oocysts induced *T. gondii* infection in all of the mice. The survival time post-inoculation with TgcatHn4 oocysts was ≥ 60 days (Table 3). *T. gondii* cysts were not detected in brain smear or histologic section at 60 DPI, but observed at 138 DPI (Figure 2B). Compared to the virulent *T. gondii* strain (Figure 2D), the pathological lesions and parasites loading of the TgcatHn4 strain of *T. gondii* in the ileum of mice were not found at 60 DPI (Figure 2C).

**Table 3:** Virulence of the oocyst of TgCatCHn4 *T. gondii* strain (ToxoDB#9) on Swiss mice by orally (60 days post-inoculation).

**No. of oocysts fed**	**No. of infections/No. of inoculations (%)**	**Days of survival^a^**	**Brain cysts**
10^5^	5/5 (100%)	≥60/5	Not found
10^4^	5/5 (100%)	≥60/5	Not found
10^3^	5/5 (100%)	≥60/5	Not found
10^2^	3/5 (60%)	≥60/5	Not found
10^1^	2/5 (40%)	≥60/5	Not found
1	1/5 (20%)	≥60/5	Not found
<1	0	≥60/5	Not found
Blank control	0	≥60/5	Not found

a *Mouse survival days/Number of surviving mice*.

## Discussion

The present study that aimed to assess the impact of *T. gondii* on captive felids faced several challenges. The first one was that the technique in handling large cats has not been well developed. The second was the limited number of serum and tissue samples. From 2015 to 2017, sick or dead felids were sent to our laboratory for etiological diagnosis, also allowed us to conduct surveys and perform specific research studies on *T. gondii*. This epidemiological study may provide clues on the process of *T. gondii* infection in felids.

In the present study, the prevalence of antibodies to *T. gondii* in felids from the zoo was higher than those from farm and pet hospital (Table 1). This difference may be related to the dietary habits or environment factors. Our survey indicated that the tigers and lions in zoos were generally fed with raw meat, most of the pet cats were given commercial cat food, and the masked palm civets in farm consumed cooked food. The possibility that raw meat contained infective *T. gondii* cysts was higher than that in well cooked meat. Raw meat could thus be a significant risk factor in felids from zoos, and considering that cooked meat is not suitable for them, then the use of pre-frozen meat may be an effective preventive measure against *T. gondii* infection. Furthermore, additional attention should be given to birds and rodents in the zoo as these may serve as intermediate hosts and mechanical transmitters of *T. gondii* oocysts.

The risk of acquiring *T. gondii* infections in adult felids was higher compared to that in juveniles. Only one giant cat (tiger: death after birth due to meconium aspiration), which was 1 day old, was determined to be *T. gondii* antibody- negative. Increasing rates of prevalence of *T. gondii* antibodies in older felids indicated postnatal exposure to *T. gondii*, which was in agreement with the results of previous study (Ramos Silva et al., 2007; Abdou et al., 2013). Gender difference was not a risk factor for toxoplasmosis in the felids examined in this survey.

Taken together, the seroprevalence of *T. gondii* in captive felids from Henan Province was 21.3% (10/47) in the present study. In Henan Province, the seroprevalence of *T. gondii* in free-range chickens was 18.9% (132/700) (Feng et al., 2016), 51.6% in cats (16/31) (Yang et al., 2015), 12.7% (99/779), and 20.7 (174/840) in domestic sheep (Zhang et al., 2016; Yang et al., 2017), 23.7% (627/2642) in pig (Wen et al., 2015), and 10.2% (20/197) in farm-reared ostriches (unpublished data). Compared to these reports, the seroprevalence of *T. gondii* was similar in captive felids from the same location, although only a limited number of samples were examined. The results of the present study were indicative of a widespread *T. gondii* infection of animals in Henan Province. This also indicated that there is a potential threat of *T. gondii* infection in humans and other animals.

Special economic animal feeding schemes have been developed for the masked palm civet as these have become a huge industry in China, particularly by using its fur, meat, and in producing “Civet coffee.” However, masked palm civets carry several pathogens that may zoonosis. Our understanding of *T. gondii* infection status in masked palm civets is limited, which speculate could shed millions of *T. gondii* oocysts via their feces. No *T. gondii* DNA was detected in masked palm civet from the Fujian zoo (Chen et al., 2015). Approximately 27.6% of masked palm civets from farms in Hainan Province were *T. gondii* seropositive; however, we have no clear information on the composition of their feed (Hou et al., 2016). Farm-reared masked palm civets were all negative for *T. gondii* (0/10) in this study. The climate between Henan Province (humid and subtropical) and Hainan Province (tropical) may have contributed to this difference. Furthermore, our results suggest that thoroughly cooked chicken meat, washed fruits and vegetables may prevent toxoplasmosis. More risk assessment of *T. gondii* should be conducted in farm-reared felids.

In China, zoos are public places where humans spend some leisure time. The oocysts of *T. gondii* that are shed by captive felids may be a risk for tourists and people working in the zoos. In 2010, 13.73% of the workers in Shenzhen zoos were seropositive for *T. gondii*, thereby rendering it as a high risk population for toxoplasmosis (Xie et al., 2010). The correlation between the risk for infection with *T. gondii* between tourists and captive felids in zoos should thus be explored in future investigation. Toxoplamosis in lions and tigers was first reported in 1989 (Dorny and Fransen, 1989; Ocholi et al., 1989). The seropravalence of captive felids in various zoos in China have been reported, including in that in Shanghai (84.2%, 16/19) (Zhang et al., 2000), Chengdu (60.9%, 14/23) (Zhao et al., 2009), Beijing (43.8%, 7/16) (Zhao et al., 2015), and Fuzhou (40.0%, 2/5) (Chen et al., 2015). In Henan Province, the *T. gondii* seropositive rate of felids in tigers and lions (88.9%) and had a high titer in MAT (Table 1), which was higher than that in other zoos from the China. The prevalence of *T. gondii* antibodies in zoo felids was 81.4% (35/43) in Mexico (Alvarado-Esquivel et al., 2013), 36.2% (51/141) (de Camps et al., 2008), and 59.3% (35/59) (Spencer et al., 2003) in United States, 15.4% (21/136) in Thailand (Thiangtum et al., 2006), 75.0% (6/8) in Portugal (Tidy et al., 2017), 69.6% (16/23) in Australia(Hill et al., 2008), and 64.9% (24/37) (Silva et al., 2001b), 54.6% (472/865) (Silva et al., 2001a), 66.7% (38/57) (Ullmann et al., 2010) in Brazil. All these higher seropositive rates for *T. gondii* in zoo felids reveal a widespread exposure to *T. gondii* in captive felids from zoos around the world.

One viable *T. gondii* strain was isolated from cat tissues in the present study, followed by a previous report from Chinese cats (Yang et al., 2015), and this strain was designated as TgCatCHn4. Genotyping of these isolates indicated that it was of the ToxoDB genotype #9 (Chinese 1) by 10 genetic makers. Although *T. gondii* infection occurs at a low level in the cats from pet hospitals (7.1%), prevented measures against toxoplasmosis should continue to be implemented. Efforts in controlling *T. gondii* infections in pet cats, including offering commercial food, inactivation of fecal *T. gondii* oocysts, and monitoring proper hygiene should be recognized. The isolation of viable *T. gondii* is the gold standard for detecting live *T. gondii* parasites. However, the success in there isolation is influenced by the density of *T. gondii* cysts in the tissues. The heart has been proven as the ideal tissue for the isolation of *T. gondii* compared to the brain or muscle (Dubey et al., 2015). However, the density and distribution of *T. gondii* cysts in tissues depend on the concentration of initial infected parasites and its virulence. Unsuccessfully isolations may be related to the low density of *T. gondii* cysts in felids from these samples. Approximately 122 viable *T. gondii* strain have been isolated from animals and humans in China. Among these, 85 *T. gondii* isolates were from cats, and 73 (85.9%) *T. gondii* isolates were genotyped as ToxoDB#9 (Dubey et al., 2007; Yang et al., 2015, 2017; Wang et al., 2016). Combined with our results, ToxoDB#9 is the predominant strain in China.

The virulence of the isolated strain TgCatCHn4 was evaluated via oral administration in outbred Swiss mice. The *T. gondii* oocysts were collected from the feces of cat. The first batch oocysts were detected on the sixth day post feeding and significantly reduced by the 11th day, which coincident with the findings of a previous report (Dubey and Prowell, 2013). The oocysts presented extremely weak pathogenicity and a lower cyst forming rate (Table 2). Furthermore, this strain showed favorable adaptive capacity in the intestines of the host, and the intestine lesions were not found at 60 DPI (Figure 2C). A growing number of research studies have shown that the same *T. gondii* genotype may exhibit different virulence and pathogenicity in the same animals. Cheng et al. (2015) reported that the ToxoDB#9 genotype in different stains has different virulence and pathogenicity rates in mice. Two ToxoDB#9 strains from sheep differences in virulence, pathogenicity, and encystation among mice, and CV-1 cells (Yang et al., 2017). Recently, Verma et al. (2017) reported that the same genotype of *T. gondii* in bobcats shared different polymorphic genes of GRA6 and GRA7. The resuslts indicated that 10 genetic makers may be not enough for identify the genotype of *T. gondii*, sequence analysis may be more accurate. Further, no evaluation standard for virulence in *T. gondii* has been established, which could facilitate in understanding the pathogenic relationships between *T. gondii* and its hosts.

In conclusion, the high prevalence of *T. gondii* infection in captive felids should be examined in more detail. Preventive measures against oocysts contamination of the environment must be further evaluated, including the use of pre-frozen meat, cleaned fruits and vegetables, monitoring birds and rodents, inactiving oocysts from feces, and proper hygiene.

## Author contributions

YY performed the data analysis and wrote the manuscript. YF performed the laboratory tests. YL, HD, TL, and YJ helped in collecting samples. XZ participated in the RFLP analysis. LZ helped in the revising of the manuscript.

### Conflict of interest statement

The authors declare that the research was conducted in the absence of any commercial or financial relationships that could be construed as a potential conflict of interest.

## Abbreviations

MAT: modified agglutination test
DPI: day post-inoculation
PCR: polymerase chain reaction.
